# Production of Conjugated Linoleic Acid (CLA) by *Lactiplantibacillus plantarum*: A Review with Emphasis on Fermented Foods

**DOI:** 10.3390/foods13070975

**Published:** 2024-03-22

**Authors:** Massimo Iorizzo, Catello Di Martino, Francesco Letizia, Thomas W. Crawford, Gianluca Paventi

**Affiliations:** 1 Department of Agricultural, Environmental and Food Sciences, University of Molise, Via De Sanctis, 86100 Campobasso, Italy; iorizzo@unimol.it (M.I.); f.letizia@studenti.unimol.it (F.L.); paventi@unimol.it (G.P.); 2 Global Agronomy, LLC, Marana, AZ 85658, USA; globalagronomy@gmail.com

**Keywords:** lactobacilli, functional food, lactic acid bacteria, dairy products, vegetable oils

## Abstract

The term Conjugated Linoleic Acid (CLA) refers generically to a class of positional and geometric conjugated dienoic isomers of linoleic acid. Among the isomers of linoleic acid *cis9*, *trans*11-CLA (*c*9, *t*11-CLA) and *trans*10, *cis*12-CLA (*t*10, *c*12-CLA) are found to be biologically active isomers, and they occur naturally in milk, dairy products and meat from ruminants. In addition, some vegetables and some seafoods have also been reported to contain CLA. Although the CLA levels in these natural sources are insufficient to confer the essential health benefits, anti-carcinogenic or anti-cancer effects are of current interest. In the rumen, CLA is an intermediate of isomerization and the biohydrogenation process of linoleic acid to stearic acid conducted by ruminal microorganisms. In addition to rumen bacteria, some other bacteria, such as *Propionibacterium*, *Bifidobacterium* and some lactic acid bacteria (LAB) are also capable of producing CLA. In this regard, *Lactiplantibacillus plantarum* (formerly *Lactobacillus plantarum*) has demonstrated the ability to produce CLA isomers from linoleic acid by multiple enzymatic activities, including hydration, dehydration, and isomerization. *L. plantarum* is one of the most versatile species of LAB and the bacterium is widely used in the food industry as a microbial food culture. Thus, in this review we critically analyzed the literature produced in the last ten years with the aim to highlight the potentiality as well as the optimal conditions for CLA production by *L. plantarum.* Evidence was provided suggesting that the use of appropriate strains of *L. plantarum*, as a starter or additional culture in the production of some fermented foods, can be considered a critical factor in the design of new CLA-enriched functional foods.

## 1. Introduction

**Figure 1:**
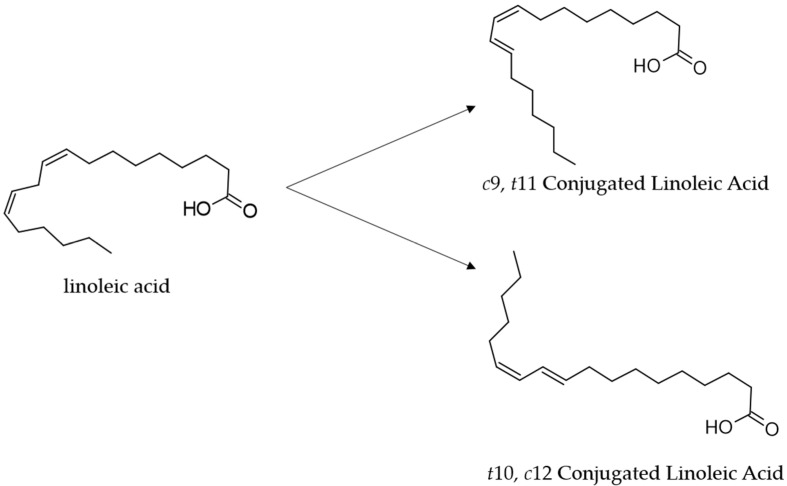
Structures of the linoleic acid (18:2) and its major conjugated isomers *cis* 9, *trans* 11-CLA (rumenic acid, CLA1) and *trans* 10, *cis* 12-CLA (CLA2).

Conjugated linoleic acid (CLA) has received increasing attention in the last two decades for its potential health benefits [1]. CLAs, some of which are polyunsaturated fatty acids (PFA) of the ω-6 series, comprise a group of positional and geometric (*cis* or *trans*) isomers of linoleic acid (LA; *cis*-9,12-octadecadienoic acid 18:2) with a conjugated double bond [2]. The isomers *cis*-9, *trans*-11(*c*9*t*11, CLA1), commonly called rumenic acid, and *trans*-10, *cis*-12 (*t*10*c*12, CLA2) are the most abundant CLA isomers, naturally present in several foods [3] (Figure 1).

CLA is primarily a product of microbial metabolism in the digestive tract of ruminants, and it ultimately accumulates in milk, beef, and dairy products [4]. CLA is also present in vegetable oils (e.g., sunflower, soybean, castor, safflower, and sesame oils) and some fish oils (salmon and lake trout) [5,6].

Potential benefits to human health are the main reason for scientific interest in CLA. Recently, several properties have been attributed to CLA including anti-carcinogenic or anti-cancer effects [7], prevention and treatment of diabetes [8], anti-atherosclerosis [9] and anti-osteoporosis effects [10], prevention of increases in body fat [11], lowering of LDL-cholesterol [12], and anti-inflammatory and immunomodulatory properties [13].

As far as the voluminous literature on CLA is concerned, to date, a definitive cause-and-effect or biochemical relationship has not been established between the consumption of the CLA isomers and the aforementioned beneficial effects [14,15,16,17,18]. Currently, scientific interest in the consumption of CLA persists, and this interest is mainly aimed at verifying the safety and efficacy of CLA on human health [19].

A review by den Harting summarizes the pre-clinical and human studies conducted using CLA to date, which collectively suggest that CLA has efficacy against cancer, obesity, and atherosclerosis [20]. 

The main interest surrounding CLA is its anti-carcinogenic (preventing the onset of cancer) or anti-cancer (diminishing or eliminating cancerous growth) effects, and some recent studies have highlighted its effectiveness [7,21,22].

Recent insights support current recommendations of public health guidelines around the world that emphasize the consumption of ω-6 fatty acids (FA) [23,24,25] and advocate a reduction in dietary saturated fatty acids (SFAs) [26,27,28]. Therefore, CLA have become an object of study for applications in food production [1]. However, the beneficial effects of CLA at low concentrations may not be significant. The recommended dietary allowance of CLA for humans ranges from 1 to 3 g/d to accomplish desired health benefits [29,30].

Natural CLA is sourced mainly from meat and dairy products, which contain 0.6–10.0 mg CLA/g of fat and 3.4–9.4 mg CLA/g of fat, respectively [31,32]. Therefore, it is clear that the CLA content in these products is too low to be able to cause a beneficial effect in consumers. Thus, it may be of interest to look for strategies such as bacterial production [33,34,35] to increase CLA content in food [36,37].

The amount of CLA in food can be increased by using various enzymatic, chemical and biological methods [35]. The most environmentally friendly method for CLA synthesis is based on microbial biosynthesis [31,38], and many lactic acid bacteria (LAB) demonstrate the ability to produce CLA isomers from LA [39,40,41].

Among LAB, *Lactiplantibacillus plantarum* (formerly *Lactobacillus plantarum*) have been identified as the most efficient CLA-producers among food-derived LAB [42,43].

*L. plantarum* is a LAB species with high ecological and metabolic adaptability [44,45,46] and is capable of inhabiting a variety of environments, including animal and human gastrointestinal tracts [47,48]. Some strains belonging to *L. plantarum* species are proposed as animal or human probiotics [49,50,51,52,53,54,55,56] and are extensively utilized as starter cultures in various fermented foods [57,58,59,60,61,62,63,64,65].

In this review, we highlight the role of *L. plantarum* in CLA production. To this aim, in the first section of the paper we described the molecular mechanisms of CLA biosynthesis in microorganisms, with a special focus on *L. plantarum*; subsequently, among the plethora of papers dealing with CLA production in LAB, we analyzed all the studies dealing with the synthesis of CLA isomers by *L. plantarum* reported in the literature (Scopus and PubMed as sources) of the last ten years. We trust that this review can provide support for the use of this bacterial species in the production of CLA-enriched foods.

## 2. Microbial Biosynthesis of CLA

**Figure 2:**
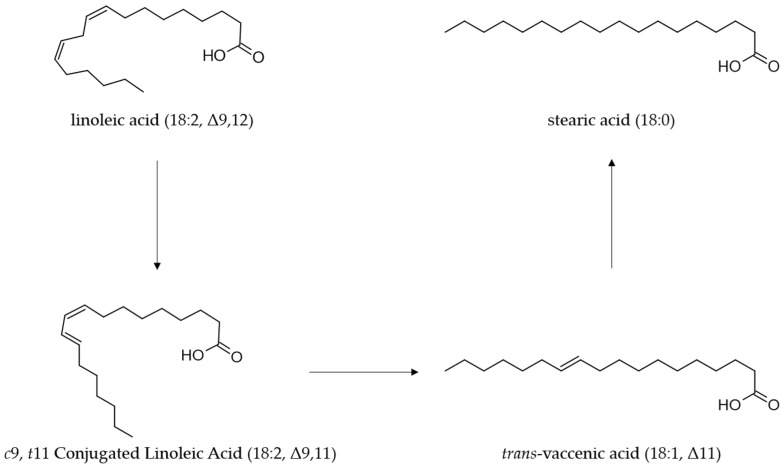
Ruminal biohydrogenation process of linoleic by *Butyrivibrio fibrisolvens*.

CLA, which is primarily a product of microbial metabolism in the digestive tract of ruminants, ultimately accumulates into ruminant-derived products such as meat, milk and other dairy products [66,67]. The PFA, present in the diet of ruminants are metabolized in the rumen by different species of microorganisms [68,69]. During this process, the PFA, including LA, are converted through isomerization and hydrogenation to stearic acid (C18:0) as the end-product [70,71]. LA is first isomerized to CLA, mainly rumenic acid (C18:2 *c*9*t*11, CLA1), and finally, through a biohydrogenation mechanism by reductase enzymes, the CLA isomers are converted to stearic acid in the rumen [72] (Figure 2).

All intermediates of this biohydrogenation process are absorbed in the gut and transported through the blood stream to different body tissues [73].

Rumen bacteria, especially *Butyrivibrio fibrisolvens*, are the initial microorganisms for CLA production, playing a pivotal role in the biotransformation and accumulation of CLA in ruminant-derived products [74]. Other bacteria such as LAB, *Bifidobacterium*, and *Propionibacterium* proved to efficiently synthesize CLA [33]. Among LAB, *Lactobacillus strains* and in particular *L. plantarum* have a high *capacity* to produce *CLA* [34,42,43,75,76,77].

It is unclear why bacteria convert LA into CLA-isomers. One of the best-supported hypotheses is that PFA such as LA are toxic to many bacteria by inhibiting cell growth, restructuring cell membranes, and interfering with biosynthesis of native fatty acids [78]. To survive such toxicities, CLA-producing bacteria may have evolved to carry out a biohydrogenation process which is the reduction of double bonds on the carbon chain, producing non-toxic SFAs as final products [33,34,79]. During biohydrogenation, various CLA isomers are formed [72,80].

The capability to produce CLA has been shown to vary among different LAB, and scientific data indicate strain-specificity in the ability to produce these isomers of LA [39,81,82].

**Figure 3:**
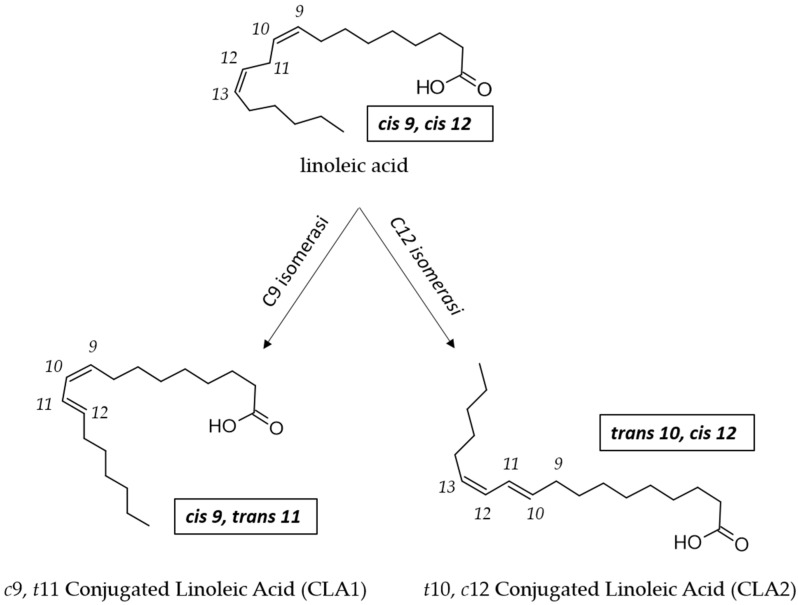
Enzymatic conversion of LA to CLA1 and to CLA2 by LA isomerase (PAI) in *Propionibacterium*.

Rumen bacteria and *Propionibacterium* transform LA to CLA by a one-step reaction catalyzed by a LA isomerase (LAI) named PAI (*Propionibacterium acnes* isomerase; Figure 3) [83]. Also in LAB, LAI activity is considered to be the key factor involved in the bioconversion of LA to CLA [34], but there is a lack of information regarding the molecular mechanism behind the ability of LAB to convert LA to CLA [73,84].

## 3. Molecular Mechanism of CLA Synthesis by *L. plantarum*

**Figure 4:**
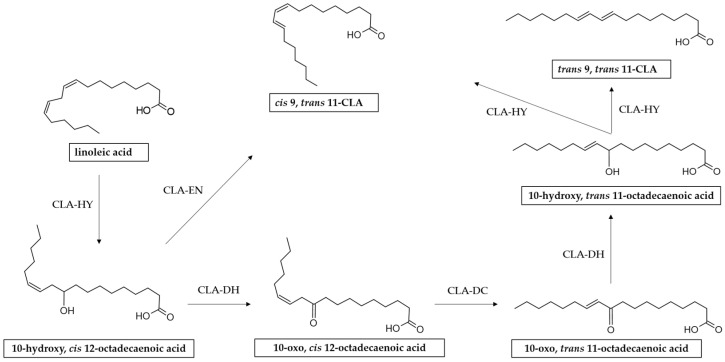
Reaction scheme of LA isomerization to CLA by *L. plantarum*. CLA-HY: linoleate hydratase, a member of the Myosin Cross Reacting Antigen (MCRA) family; CLA-DH: short-chain hydrogenase/oxidoreductase; CLA-DC: acetoacetate decarboxylase.

It has been reported that the process of CLA biosynthesis by *L. plantarum* is much more complicated [85]. Some studies have indicated that the mechanism for CLA production by *L. plantarum* involves multiple reactions including hydration, dehydration, and isomerization catalyzed by three enzymes, i.e., CLA hydratase (CLA-HY), CLA short-chain dehydrogenase (CLA-DH), and CLA acetoacetate decarboxylase (CLA-DC) [73,86,87,88,89] (Figure 4).

These enzymes are separately encoded from three genes: CLA-HY is encoded by lp_0139 (*cla-hy*), while CLA-DH and CLA-DC are encoded by lp_0060 (*cla-dh*) and lp_0061 (*cla-dc*), respectively [90,91].

Liu and others showed that strains of *L. plantarum* with different CLA biosynthesis abilities possessed different transcriptional levels of *cla-hy*, *cla-dh*, and *cla-dc*, suggesting that the upregulation of the CLA yield may be achieved by regulating the transcription of these genes [91]. In another study, it was found that these three genes in *L. plantarum* WCFS1 are regulated by a LysR-type transcriptional regulator (LttR) [92]. LttR family proteins regulate a diverse set of genes, including those involved in stress responses, motility, virulence, amino acid metabolism, quorum sensing and motility [93]. In another recent study on *L. plantarum* AR195, it was shown that in addition to LttR, the arginine repressor ArgR2 positively regulated *cla-dh* and *cla-dc* transcription [92].

In addition to the complex enzyme system described above, in a study conducted by Ortega-Anaya and others it was demonstrated that *L. plantarum* CFQ-100 (subculture of *L. plantarum* ATCC 8014) possesses a multifunctional protein belonging to the enolase family (α-enolase). In addition to having a central role in glycolytic metabolism, α-enolase has a collateral role in the biohydrogenation metabolism of LA, being capable of catalyzing the formation of 9-*cis*, 11-*trans*-CLA through dehydration and isomerization 10-hydroxy-12-*cis*-octadecenoic acid [94].

## 4. CLA Production by *L. plantarum* in Different Culture Media

The ability to synthesize CLA not only differs among different bacterial species, but also between different strains belonging to the same species [39,81,82,84,95]. In addition, CLA production can be affected by several factors, such as added LA concentration, pH, temperature, and fermentation time [40,41,82,96]. In this context, for each individual bacterial strain, the optimization and standardization of fermentation conditions are necessary to maximize the production of CLA isomers.

The influence of the processing conditions to produce CLA in vitro using *L. plantarum* has been largely studied. The main results obtained in several studies on CLA production by several *L. plantarum* strains in different culture media with LA or ricinoleic acid (RA; 12-hydroxy-*cis*-9-octadecaenoic acid 18:1) as a CLA source are summarized in Table 1.

Temperature is an important physical factor that can influence the growth of *L. plantarum*, a mesophilic bacterium that has an optimal growth temperature of approximately 37 °C [111]. In most of the studies cited below, the optimal conditions for maximum CLA production of *L. plantarum* were between 30 and 40 °C. It has been reported that regulating the temperature can induce variations in the lipid composition of microorganisms, resulting in the maintenance of the integrity of the cell membrane [112]. Therefore, temperature appears to be a critical factor in CLA production since LAI in *L. plantarum* was found to be a multi-component enzyme system widespread in both the soluble and the membrane fractions [86].

However, optimal temperatures for bacterial growth and CLA production do not always coincide. In fact, in a study conducted by Devi and Rashmi, the maximum growth of *L. plantarum* ATCC 8014 was observed at 37 °C, but the highest rate of CLA production was detected at 40 °C [98]. In other studies, maximum CLA production was achieved at 30 °C after 48 h [75,103], while two reports recorded the highest CLA production after 24 h at 37 °C and 40 °C, respectively [99,108]. As highlighted in these studies and discussed more later, in addition to temperature, fermentation time is another important factor that determines the growing phase of LAB and indirectly reflects the synthesis of LAI [113].

In general, all studies have shown that the production of CLA increases over time until it reaches a maximum, after which the amount of CLA tends to decrease progressively [98]. Therefore, the maximum amount of CLA can be obtained at a specific time for each individual strain, according to the different phases of bacterial growth (exponential and stationary phases), as well as LA concentration and LAI activity [2].

During fermentation, the pH of the medium significantly affects the shape and function of proteins, including enzymes responsible for the metabolic processes of fermentation. Each enzyme has an optimal range of pH, and the pH of fermentation outside of this range is associated with reduced enzyme activity. LAI is sensitive to pH and it has been shown that ruminal pH between 6.0 and 7.0 was associated with a high production of CLAs in rumen cultures [114].

All studies mentioned above indicate the significance of a range of pH around neutral for the synthesis of CLA from *L. plantarum*. This optimal pH range appears to result from a compromise between the optimal pH for the growth of *L. plantarum* [115] and the optimal pH for the activity of the key enzymes involved in the biosynthesis of CLA. The optimal initial culture pH for the production of CLA by different *L*. *plantarum* strains has been reported as 5.5 [104] and 6.0 [106,116,117], while Ando and others found that the maximum CLA synthesis from *L. plantarum* JCM 1551 occurred at pH 6.5 [100]. As stated above, CLA synthesis in *L. plantarum* includes the enzymes CLA-HY, CLA-DH and CLA-DC. In a study conducted by Takeuchi and others, it was found that the CLA-HY enzyme is optimally active at pH 5.5 [118].

In addition to pH and temperature, the substrate concentration (LA) is also crucial for the production of CLA. The LA probably inhibits growth by increasing the permeability of the bacterial membrane as a result of its surfactant action [119].

The amount of CLA in biomass depends on the initial LA concentration, cell growth state, and LAI activity for the bioconversion of LA to CLA [80]. As a general consideration, it can be said that CLA production increases with the increase in concentration of LA [78,97,104,106], provided that the concentration of LA does not exceed a tolerable limit. However, as we will see in some of the studies cited below, individual strains of *L. plantarum* differ in their tolerance to the initial concentration of LA.

Many LAB, including *L. plantarum*, demonstrate the ability to produce CLA isomers from the LA, and those isomers are mainly CLA1 and CLA2.

The washed cells of *L. plantarum* ZS2058, isolated from Chinese traditional fermented vegetables, in de Man–Rogosa–Sharpe (MRS) medium, containing 0.5 mg/mL of LA, produced a mixture of CLA1 and CLA2, 96.4% of which was CLA1. After 24 h at 37 °C under aerobic conditions, 312.4 μg/mL of CLA1 was produced [109].

In a subsequent work conducted by the same research group, it was found that the optimal pH and optimal temperature of bioconversion by *L. plantarum* ZS2058 yielding CLA were 6.5 and 40 °C, respectively [108]. Natural sauerkraut, a fermented food made primarily from fermentations of cabbage, contains a great number of LAB including *L. plantarum*, which is often predominant [120,121]. In a study reported in 2009 [105], fifteen CLA-producing LABs were isolated from natural fermentations of sauerkraut. In MRS 2.5 mL/L of LA was added and after 24 h at 30 °C *L. plantarum* NCUL005 showed the highest CLA-producing ability (0.623 mg/mL). The transformation efficiency of converting LA into CLA by NCUL005 was 26.67%, and the CLA produced by *L. plantarum* NCUL005 comprised a mixture of 32.2% CLA1 and 67.8% CLA2 isomers [105].

*Lactobacillus plantarum* WU-P19 isolated from a sample of a traditional fermented Indian trumpet (midnight horror, *Oroxylum indicum*) was investigated, with the aim of enhancing the LA conversion to CLA. Under static conditions at 37 °C and pH 6.0, after 36 h, MRS was supplemented with the cell permeabilizing agent chitosan, resulting in an increased cellular uptake of LA (37 mg/g) and production of 21 mg/g total CLA. Nearly 50% of total CLA was CLA1 and the remainder was CLA2 [106].

In a study conducted by Liu and others, forty-three LAB strains with a CLA-producing ability were isolated from three naturally fermented pickle brines [103]. At 48 h in MRS broth to which LA was added (100 µg/mL), *L. plantarum* lp15 exhibited the greatest capacity to produce CLA (26.1 μg/mL) and the highest tolerance to LA, up to 600 µg/mL. This strain converted about 25% of LA into CLA isomers, of which 75% was CLA1 [103].

Yang and others assessed the capability of some strains of food-derived lactobacilli to produce CLA from LA. They found that *L. plantarum* ZS2058 was the most efficient CLA producer in MRS broth with more than 50% LA conversion to CLA1 and *t*9, *t*11-CLA as dominant isomers [110].

The ability of different LAB species to produce CLA from LA has also been evaluated [99]. After 24 h at 37 °C in MRS broth, containing 1 mg/mL of LA and 1 mg/mL Tween 80, *L. plantarum* DSM 20179 showed the highest potential to produce CLA (95.25 μg/mL). An optimization analysis also showed that the maximum CLA production (240.69 μg/mL) by *L. plantarum* DSM 20179 can be obtained in skim milk (SM) supplemented with 1 mg/mL Tween 80, 7 g/L D-glucose, 3.0 mg/mL LA and 4.01 g/L yeast extract [99].

Sixty-four strains of food-grade lactobacilli and bifidobacteria were examined to verify their ability to produce CLA [41]. Lactobacilli were grown in MRS medium, and LA was added (500 μg/mL) at 37 °C for 48 h. In this case, more than 90% of the CLA was detected in the supernatant. *L. plantarum* CRL1920 isolated from chicha (fermented maize) and *L. plantarum* CRL1935 isolated from cheese, were able to conjugate LA with a conversion rate of 3.47% and 3.50%, respectively. In detail, *L. plantarum* CRL1920 and *L. plantarum* CRL1935 produced 6.95 and 7.26 μg/mL of CLA1; 5.11 and 5.22 μg/mL of CLA2; and 5.28 and 5.04 μg/mL of *trans*-9, *trans*-11 CLA, respectively [41].

Fifty-seven CLA producing LAB strains isolated from fermented dairy products were screened for their ability to produce CLA in MRS broth and SM, supplemented with 0.5 mg/mL of linoleic acid [95]. Positive strains were classified as *L. plantarum* (44%), *L. gasseri* (30%), *L. fermentum* (21%) and *L. salivarius* (5%) species. *L. plantarum* HIF15 was reported as the best producer of CLA (46.18 µg/mL in MRS and 52.61 µg/mL in SM) with a higher amount of CLA1 isomer (34.73 µg/mL in MRS; 38.31 in SM) [95].

Ribeiro and others conducted a study on 110 LAB isolated from a traditional Azorean cheese to test their ability to convert free LA to CLA. *L. plantarum* L2C21E8 and *L. plantarum* L3C1E8 were selected as CLA-producing strains. LABs were incubated in MRS broth containing free LA (0.5 mg/mL) and 2% (*w*/*v*) Tween 80, at 30 °C for 48 h. Preliminarily, CLA production was quantified by a spectrophotometric method [102]. *L. plantarum* L2C21E8 and *L. plantarum* L3C1E8 produced 17.94 and 15.36 μg/mL of CLA (expressed as CLA1 concentration) with a conversion of 3.59% and 3.07%, respectively. Afterwards, the CLA profiles were determined in cell supernatant and in cell pellet using gas chromatography–mass spectroscopy (GC–MS). CLA1 and CLA2 were the most abundant isomers generated, and they were mainly found in the cell supernatant.

These results are in agreement with previous studies that have shown that the production of CLA is located primarily in the extracellular *phase* [41], although it can also be found in smaller amounts in the cell membrane as a structural lipid [122].

In a study reported in 2009, six different strains of *L. plantarum* were examined for their ability to synthesize different metabolites including CLA [123]. *L. plantarum* strains were grown in MRS medium containing LA from 1% to 10% (*w*/*v*), and the LA metabolites formed in the medium were identified and quantitated by GC–MS. *L. plantarum* 2–3 showed maximum growth and conversion of LA to different metabolites from 1% to 10% (*w*/*v*) of LA supplied. The production of total LA metabolites gradually increased with the increase of LA concentration from 1% to 10% (*w*/*v*) [123]. This indicates that the *L. plantarum* showed high tolerance to LA by converting it into less toxic compounds. As previously reported, in fact, other studies have also suggested that the conversion of free LA metabolites to CLA could be a detoxification mechanism adopted by bacterial cells [33].

The aim of a subsequent study conducted by Aziz and others was to investigate the ability of *L. plantarum* YW1 to produce CLA. The results showed that *L. plantarum* YW11 is able to convert LA into a CLA isomer (rumenic acid) and stearic acid, at different concentrations of LA (also in this case1–10% *w*/*v*) [107]. *L. plantarum* YW11, isolated from kefir from Tibet, has been found to possess antimicrobial, anticancer, antioxidant, and immuno-regulatory activities [124,125].

In another study, some probiotic properties were evaluated using 10 strains of high CLA-producing LAB isolated from *Jeotgal* (a high-salt fermented seafood) [75]. The LAB were cultured in MRS broth containing LA (5 mg/mL) and 1% (*v*/*v*) Tween 80 at 30 °C for 48 h. The CLA isomers were quantified using GC–MS. *L. plantarum* JBCC105683 produced the highest concentration of CLA (748.8 μg/mL) and the ratio of CLA1 to CLA2 was approximately 80:20, whereas *L. plantarum* JBCC105645 produced an approximately equal proportion of CLA1 and CLA2 (~50:50 ratio). *L. plantarum* JBCC105683 strongly stimulated the immunological regulatory gene PMK-1 and a host defense antimicrobial peptide gene, *clec-60*, in *Caenorhabditis elegans* and produced significant induction of tumor necrosis factor-α, interleukin (IL)-1β, IL-6, IL-12, and IL-10 in RAW 264.7 macrophages, indicating that they are good candidates for probiotics with a high CLA-converting activity [75].

Based on the results reported in this section, it is possible to highlight the following summary considerations:

- the optimal temperature for CLA production is between 30 and 37 °C, a temperature range that also represents an optimal condition for the development of a mesophilic bacterium as *L. plantarum*;
- conditions of the culture medium close to neutrality (pH 6–6.5) allows for the maximum amounts of produced CLA, with this optimal pH range resulting from a compromise between the optimal pH for the growth of *L. plantarum* and the optimal pH for the activity of the key enzymes involved in CLA biosynthesis;
- the produced amount of the various CLA isomers showed a high variability among the different *L. plantarum* strains;
- CLA production increases with the increase of concentration of LA provided up to a value of LA concentration not exceeding the tolerable limit for the bacterium, with this limit proved to depend on the strain.

## 5. CLA Production from Vegetable Oils by *L. plantarum*

**Figure 5:**
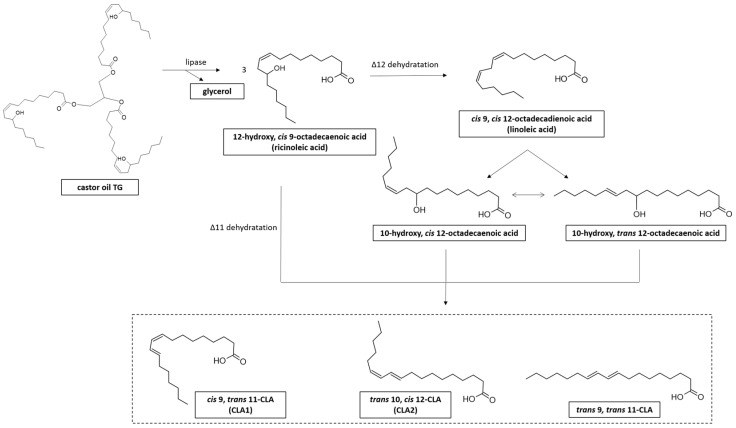
Synthesis of CLA from castor oil and ricinoleic acid (RA) derivative. RA is the most abundant (around 90%) fatty acid (FA) present in castor oil triglyceride (TG), contained in castor bean (*Ricinus communis* L.) [127].

Castor, sunflower, safflower, and sesame oils are the most common vegetable oils used as a microbial substrate for CLA production [40] (Table 2). LAB can produce CLA from RA via its direct conversion into CLA through dehydration or through transformation of RA to LA followed by isomerization of LA to CLA (Figure 5) [126].

Based on these considerations, some authors have used castor oil as an alternative substrate for the production of CLA by *L. plantarum.* The lipase enzyme for castor oil hydrolysis has been used to release RA as a substrate for CLA production [42,100,116,128].

**Table 2:** CLA production from vegetable oils by *Lactiplantibacillus plantarum* (formerly *Lactobacillus plantarum*).

*L. plantarum* Strain	Culture Medium, Environmental Conditions	CLA Source	Total CLA	CLA Isomers (%)	Ref.
AB20-961 (DSM2601)	sausage fermentation 79 h (73 h)	5% SAO	4.1 mg/g fat (7.5 mg/g fat) in sausage	n.d.	[129]
AB20-961	subculture in MRS, 37 °C, 24 h sucuk fermentation 12 h	2% HSO	6.1 mg/g fat in sucuk	69% CLA1 31% CLA2 in sucuk	[117]
AKU1009a	KPB, pH 6.5, 37 °C, washed cells, 24 h	4.0 mg/mL CO (88% RA, 5% LA, 7% other)	1.14 mg/mL	17% CLA1 83% *t*9*t*11-CLA	[130]
ATCC8014	MRS, 37 °C, pH of 6.5, 72 h, 2% washed cells	8 mg/mL SO	0.8 mg/mL	48% CLA1 52% CLA2	[78]
IMAU60042	MRS, 37 °C, 20 h	1.2 μg/mL SO (LA 67.3% of total FA)	48.7 μg/g	n.d.	[131]
JCM 1551	1.0 M citrate buffer 37 °C, pH of 6.0, 99 h	5.0 mg/mL CO	2.7 mg/mL (up to 7.5 mg/mL with 30 mg/mL CO, 171 h)	26% CLA1 74% *t*9*t*11-CLA	[116]
Lp in co-culture with *L. acidophilus*	SM, pH 6.4, 36 °C, 48 h 5% inoculum	5% SAO	316.5 μg/mL	n.d.	[132]
*L. plantarum* from buffalo milk	KPB, pH 6.5, 37 °C, 20 h 12% (*w*/*v*) washed cells, lipase	8 mg/mL CO	406 μg/mL	56% CLA1 44% CLA2	[128]
P1	MRS, 37 °C, 24 h	10 mg/mL SO	400 μg/mL	n.d.	[133]
P1201	soy-powder hydrolyzed milk 35 °C, 48 h	1% SAO	1.3 mg/g	92% CLA1 8% CLA2	[134] [135]
PTCC1058	KPB, pH 6.5, 37 °C, 121 h 15% (*w*/*v*) washed cells, lipase	4.6 mg/mL CO	1.7 mg/mL	44% CLA1 46% CLA2	[136]
PTCC1745	KPB, pH 6.5, 37 °C, 121 h 15% (*w*/*v*) washed cells, lipase	9.6 mg/mL CO	1.6 mg/mL	41% CLA1 55% CLA2	[136]
UALp-05 in co-culture with of *L. lactis* ssp. *lactis* and *L. lactis* ssp. *cremoris*	91% milk fat to produce cheddar cheese	9% SAO	75 μg/g in 90 days ripened cheese	25% CLA1 20% CLA2 20% *c*9*c*11-CLA 18% *t*9*c*11-CLA 16% *c*10*t*12-CLA	[43]

Abbreviations: CLA1, *cis*-9, *trans*-11 CLA; CLA2, *trans-*10, *cis-*12 CLA; CO, castor oil; FA, fatty acids; HSO, hydrolyzed sunflower oil; KPB, potassium phosphate buffer; LA, linoleic acid; MRS, de Man, Rogosa and Sharp medium; RA, ricinoleic acid; SAO, safflower oil; SM, skim milk; SO, sunflower oil; n.d., not determined.

More than twenty years ago, Kishino and others conducted the first study on the biosynthesis of CLA from castor oil and RA by *L. plantarum* [130]. Using washed cells of *L. plantarum* AKU 1009a, 1.14 mg/mL of CLA were produced from 4.0 mg/mL castor oil in the presence of lipase. The CLA produced was a mixture of CLA1 (0.19 mg/mL) and trans-9, trans-11 octadecadienoic acids (0.95 mg/mL) [130].

In a separate study, *L. plantarum* JCM 1551 produced 2.4 mg/mL of CLA consisting of a mixture of two isomers, CLA1 (21% of total CLA) and *trans*-9, *trans*-11-octadecadienoic acid (79% of total CLA) [100].

In a subsequent study, the same research group found that a mixture of two isomers, CLA1 and *trans*-9, *trans*-11-octadecadienoic acid, was obtained by using as catalyst washed cells of *L. plantarum* JCM 1551 in the presence of lipase and 5.0 mg/mL of castor oil, 2.7 mg/mL of CLA was produced in 99 h, and from 30 mg/mL of castor oil, 7.5 mg/mL of CLA was produced in 171 h [116].

In another study, castor oil was used as substrate for the production of CLA using washed cells of *L. plantarum* strains and lipases as catalysts [128]. Mass spectral analysis showed that CLA1 (56.55%) and CLA2 (43.45%) isomers were produced [128].

In other research, CLA was produced from castor oil using washed cells of *L. plantarum* PTCC 1058 and *L. plantarum* subsp. *plantarum* PTCC1745 [136]. *L. plantarum* PTCC1058 has been distinguished by its ability to produce extracellular 1.7 g/L CLA from 4.6 mg/mL of castor oil. The resulting CLA was a mixture of CLA1 (44% of total CLA) and CLA2 (46% of total CLA) [136].

In the above studies, in which castor oil was used, the ability of *L. plantarum* to produce CLA isomers (mainly CLA1, CLA2, and *trans*-9, *trans*-11-octadecadienoic acid) from the RA was highlighted. This capacity has been optimized by adopting a temperature of 37 °C and a pH of the growing medium ranging between 6 and 6.5 (see Table 2 for refs).

It is important to point out that the castor oil seed is one of the well-known oil seeds in some areas of Africa where it forms an important part of the diet. Among traditional condiments used in the eastern part of Nigeria are Ogiri-Igbo and other Ogiri foods that are fermented products of *Ricinus communis* [137]. Ogiri foods have played major roles in the diets of communities in rural regions, serving not only as a nutritious non-meat protein substitute, but also as condiments and flavoring agents in soups and sauces [138].

Castor oil derived from castor beans contains toxic compounds such as ricin, a Type II ribosome-activating protein, and other related compounds as ricinine and ricinoleic acid. For this reason, it cannot be consumed directly but must be processed by fermentation to remove these toxic constituents [139]. Various studies of physical, chemical, and biological treatments have been conducted to establish efficient methods for castor meal detoxification [140]. LAB fermentation has been shown to be a technique that leads to a complete detoxification of castor oil and improves its nutritional value [141]. Additional research has shown that some LAB species, including *L. plantarum,* are part of the microbial community present during spontaneous fermentation of castor oil bean in Ogiri foods [142,143,144].

In developing products containing castor oil with improved quality and safety, the inclusion of pro-technological and probiotic microorganisms (such as *L. plantarum*) is crucial and microorganisms will serve as sustainable interventions for the development of African-specific starter cultures [145]. Therefore, it is desirable that in the future scientific investigations be conducted to select CLA-producing strains of *L. plantarum* able to perform a detoxification of fermented castor oil.

Sunflower (*Helianthus annuus* L.) is considered to be one of the most important oil plants having 22–55% oil content. Sunflower oil contains approximately 15% SFA and 85% unsaturated fatty acids (UFAs), and sunflower oil UFAs have been shown to consist of 14–43% oleic acid and 44–75% LA [146].

The following studies include investigations of sunflower oil, soy milk, castor oil, cod liver oil, flax oil, and linseed oil as sources of LA for the production of CLA by LAB [78,117,131,133].

In a study conducted by Li and others, six strains of *L. plantarum* (IMAU60042, IMAU60171, IMAU10156, IMAU30126, IMAU70089, and P8), after being isolated from traditional naturally fermented dairy products, were able to convert LA to CLA using sunflower oil as a substrate or during soy milk fermentation [131]. Sunflower oil used in this experiment contained LA that was 67.3% of total FA. Soymilk was added with 6.5% sucrose and after inoculation of LABs was incubated at 42 °C. The results showed that the six *L. plantarum* strains had different abilities to produce CLA. After 20 h at 37 °C, *L. plantarum* IMAU60042 produced the highest concentration of CLA (48.7 μg/g) in MRS supplemented with 1.2 μg/mL of sunflower oil. The same strain also produced the highest concentration of CLA in soy milk (122.4 μg/g) for 12 h. The CLA was composed of CLA1 and CLA2 isomers [131].

Hosseini’s research verified the production of CLA by *L. plantarum* ATCC 8014 from sunflower oil and castor oil as cost-effective substrates, compared to linoleic acid. The reaction mixture contained 1 mL of 100 mM potassium phosphate buffer (pH 6.5), 2% washed cells and different concentrations (1, 4, 8, 12 mg/mL) of LA, sunflower oil, and castor oil. The tests were carried out micro-aerobically at 37 °C for 72 h. The washed cells of *L. plantarum* ATCC 8014 produced the highest concentration of CLA isomers, compared to other LAB species examined. Analysis of the results revealed that the CLA produced was a mixture of two bioactive isomers including CLA1 (0.38 mg/mL) and CLA2 (0.42 mg/mL) from 8 mg/mL sunflower oil [78].

Al-Saman and others investigated the impact of oil type on the production capacity of CLA through eight LAB strains belonging to different species, including *L. plantarum* P1. Two vegetable oils (sunflower oil and linseed oil) and cod liver oil were used as substrates in MRS medium containing 1% of Tween 80. The oils were added in a concentration of 10 mg/mL to the medium and incubated for three days at 37 °C. CLA produced by *L. plantarum* P1 was 400.32 μg/mL from sunflower oil, 432.55 μg/mL from cod liver oil, and 488.12 μg/mL from flax oil. Based upon the results obtained, it can be deduced that the differences in CLA production may be due to the different fatty acid composition of the oils used [133]. The oil of *Acer truncatum* Bunge (ATB) seed is a novel, edible oil with a richer content of oleic and linoleic acids than other edible oils including rapeseed, peanut, grape and sunflower oils [147]. ATB is a tree species native to China [148].

In 2017, Chen and others published a study in which they developed a new method to produce two isomers of CLA from ATB-seed oil by fermentation using *L. plantarum* CGMCC8198. A novel probiotics strain *L. plantarum* CGMCC 8198 was inoculated (1%) in MRS broth with or without 0.5 mg/mL ATB-seed oil and then incubated anaerobically at 30 °C with a gaseous mixture of 80% nitrogen, 10% carbon dioxide, and 10% hydrogen. Analyses by GC–MS showed that the concentration of CLA1 and CLA2 in ATB-seed oil could be increased by about 9- and 2.25-fold, respectively, after being fermented by *L. plantarum* CGMCC 8198 [149].

## 6. CLA-Producing *L. plantarum* Strains in Fermented Food

The management of the CLA content in foods provides an important way to increase their nutritional and functional value and may significantly improve marketing, and possibly sales, by adding value to traditional products. In the absence of added CLA-producing bacteria, CLA is mostly found in the fatty meat and dairy products of ruminant animals and is derived from the metabolism of ruminal microorganisms [150].

The estimated daily human CLA intake ranges from 200 to 1000 mg per day [151,152,153]. A dietary increase in LA in the feed of dairy cows is one of the feeding strategies to increase the concentration of CLA in milk. The main sources of LA for animal feed are mainly cereals, oilseeds, and oils [74,154]. Because the natural concentrations of CLA in milk products are relatively low (normally ranges between 2 and 37 mg/g fat) to exert their health benefits [155], production of CLA by LAB can be achieved by microbial cultures to produce functional and fermented food products containing a higher amount of CLA [31].

Below and in Table 3, we report some results obtained using different CLA-producing strains of *L. plantarum* as added cultures in different fermented dairy and meat products.

Ye and others tested the ability of *L. plantarum*, *Lactobacillus acidophilus* and *Streptococcus thermophilus* strains to produce conjugated linoleic acid (CLA) [132]. LAB were co-cultured in a medium containing skim milk supplemented with hydrolyzed safflower oil. More CLA was produced by co-culture than by using a single strain. Maximal CLA production (316.52 μg/mL) was obtained with an *L. acidophilus*–*L. plantarum* co-culture after 48 h at pH 6.4 and 36 °C [132].

The objective of another study was to examine the ability of different LAB strains isolated from artisanal cheese for their ability to produce CLA in skim milk and in simulated gastrointestinal conditions [156]. *L. plantarum* J25 bacteria were able to survive in simulated gastrointestinal conditions and to adhere to the intestinal mucosa. In skim milk from 2% LA, *L. plantarum* J25 produced 71.5% (13.72 μg/mL) CLA1 and 28.5% (9.81 μg/mL) CLA2 isomers. The tests were conducted at 37 °C for 48 h under aerobic conditions. In simulated intestinal juice solution containing 0.2% LA, *L. plantarum* J25 produced 15.05 μg/mL of CLA [156].

In our opinion, this result is of relevant interest, since it strongly suggests that potential probiotic *L. plantarum* strains preserve their ability to produce CLA even after having passed the gastro-intestinal tract; in this way, they could be able to express in situ its “postbiotic” capacities using CLA precursors contained in food.

In a study on 129 LAB strains [101], previously isolated from raw-milk, artisanal cheeses [158], *L. plantarum* L188 and *L. plantarum* L200 were recognized as producers of CLA. Miniature cheeses made with the addition of the *L. plantarum* L200 showed a higher content of CLA1 compared to the CLA1 content of the control cheeses, at 1.09% and 0.69% of total FAs, respectively [101]. Therefore, the authors of the present study suggest that the *L. plantarum* L200 strain could be used as an additional culture to increase the CLA content in cow’s milk cheeses.

A recent study investigated the characteristics of CLA-enriched cheddar cheese obtained using *L. plantarum* UALp-05 as a starter and safflower oil as a substrate for CLA synthesis [43]. The results obtained showed that *L. plantarum* UALp-05 and safflower oil did not negatively affect the composition of the cheddar cheese, contributing to a cheese in which the concentration of CLA increased even during the entire ripening phase.

Meat from ruminants generally has higher levels of CLA than does meat from non-ruminants. The highest CLA concentrations were found in lamb (4.3–19.0 mg/g lipid) and with slightly lower concentrations in beef (1.2–10.0 mg/g lipid) [152]. There is an increasing demand for meat and meat products with higher levels of polyunsaturated fatty acid (PUFA).

This also applies to CLA; in fact, even though animal source foods such as beef and dairy products naturally contain CLA, the concentration of CLA is generally considered low, especially in beef [159]. Therefore, studies investigating the enhancement of the concentration of CLA in meat and meat products by dietary manipulation and direct addition have increased in recent years [160]. LAB are a microbial group that contributes to the definition of the qualitative and sensory characteristics of fermented sausages [161,162]. *L. plantarum* has been shown as to be a dominant bacterium in many traditionally fermented sausages worldwide and is often proposed as a starter in the production of these products [57,163]. However, there are few studies related to enhancing microbial production of CLA by *L. plantarum* in the meat system [96,117,164].

Sucuk is a fermented dry and spicy sausage which is consumed in several Balkan, Middle Eastern and Central Asian cuisines [117]. Özer and others have used two CLA-producing strains of *L. plantarum* as starters in the fermentation of Sucuk. Preliminarily, twenty-three *L. plantarum* strains were screened in vitro for their ability to convert the LA of hydrolyzed sunflower oil (HSO) to CLA. The highest CLA production was obtained, after incubation at 37 °C for 24 h in MRS broth at pH 6.0 with 2% HSO, using *L. plantarum* AA1-2 and *L. plantarum* AB20-961 isolated from human sources. *L. plantarum* AB20-961 produced greater quantities of CLA isomers (CLA1 and CLA2) in sucuk during the first 24 h of fermentation, after beginning the fermentation initially at pH 5.8 or pH 6.0. In sucuk obtained using *L. plantarum* AB20-961 as starter, total CLA content increased from 4.9 to 5.4 mg/g of fat at initial pH of 5.8, and increased from 4.8 to 6.1 mg/g of fat at initial pH of 6.0. *L. plantarum* AA1-2 was not able to produce CLA during sucuk fermentation [117].

At the 63rd International Congress of Meat Science and Technology, results were presented regarding the use of *L. plantarum* AB20-961 and *L. plantarum* DSM2601 as starter cultures in sausage fermentation to enhance CLA contents of the final product [165]. These results showed that the CLA content of the sausage increased significantly during fermentation by both *L. plantarum* strains. While the CLA content of the sausage dough was 3.41 mg CLA/g fat, after the fermentation process, CLA contents of the sausages produced with *L. plantarum* AB20-961 and *L. plantarum* DSM2601 were 4.15 mg CLA/g fat and 7.54 mg CLA/g fat, respectively [165].

The aim of a subsequent study conducted by the same group [164] was to use optimized processing conditions for *L. plantarum* AB20-961 and *L. plantarum* DSM 2601 to obtain the highest CLA contents of semi-dry, fermented sausages. Results indicated that the CLA concentrations of the sausages were increased 21% by *L. plantarum* AB20–961 and 121% by *L. plantarum* DSM2601 after fermentation, compared to the initial concentration of CLA [164].

A follow-up study conducted by the same investigators determined the optimal pH, time, temperature, variety and concentration of the added dietary acid, and the initial bacterial cell density (*L. plantarum* AB20-961 and *L. plantarum* DSM2601) to maximize CLA production in fermented ground beef [96]. Using safflower oil, the greatest concentrations of CLA produced by *L. plantarum* AB20-961 and *L. plantarum* DSM2601 were 7.91 and 38.31 mg CLA/g fat, respectively [96].

As evidenced by these studies, different factors can affect the ability of *L. plantarum* to produce CLA in meat and meat products. Some of these factors include the pH of the meat, the fermentation time and temperature, the amount and variety of the FA added, and the initial starter cell density. The results obtained in the above-mentioned studies conducted by Özer and others have shown that it is possible to have microbial production of CLA during the fermentation of meat products using *L. plantarum* as a starter culture.

Soybean, *Glycine max* (L.), is an important crop that serves as a significant source of lipids and proteins, and soybean is the most commonly produced oil crop in the world. Soybean oil is primarily used in the production of shortening, margarines, cooking or frying oils, salad dressings, and mayonnaise. Soybeans, in addition to their nutritional value, contain specific bioactive phytochemicals [166,167] and approximately 18–24% of total lipids. The FA in soybean seed oil include palmitic acid (11%), stearic acid (4%), oleic acid (23%), LA (54%), and α-LA (8%) [168]. Due to their high LA content, soybeans have the potential to produce CLA-rich foods through LAB fermentation [2]. In a study reported in 2015 [157], *L. plantarum* S48 and *L. plantarum* P1201 produced CLA1 and CLA2 isomers from 8% skim milk medium supplemented with three different free LA concentrations (0.25, 0.5 and 1 mg/mL) at 35 °C for 48 h. Subsequently, the authors conducted comparative tests on the production of CLA in 10% fresh, steamed, and roasted soy-powder milk. After 48 h of fermentation at 37 °C, *L. plantarum* S48 and *L. plantarum* P1201 produced the greatest amounts of total CLA in steamed soy milk powder, i.e., 183.57 μg/mL and 198.72 μg/mL, respectively, of which 165 μg/mL and 180 μg/mL were CLA1. In other subsequent works, it has been shown that soy milk fermented by CLA-producing *L. plantarum* P1201 is enriched with both CLA and flavonoids and possesses some functional properties such as antioxidant activity and positive modulation of lipid metabolism [134,169].

Hwang and others conducted a study investigating the production of fermented soy milk using *L. plantarum* P1201. Soymilk with 2% sucrose was hydrolyzed with 10 U of cellulase, protease, and esterase at 37 °C for 24 h, and finally 1.0% safflower oil and the starter (2.0 × 10^7^ CFU/mL) were added. Fermentation was carried out at 35 °C for 48 h. *L. plantarum* P1201 increased the content of isoflavones in the aglycone form (daidzein, glycitein and genistein) and produced CLA in fermented soymilk by improving some functional properties that positively influenced adipogenesis and lipid metabolism [135].

## 7. Conclusions

Thanks to the growth of scientific evidence, interest in the biological significance of CLA to human nutrition continues to increase. At present, different functional foods such as yogurt, cheese, and fermented soya milk, are manufactured with CLA-producing bacteria in order to obtain a final product with high CLA content. Although in vitro production of CLA has been intensively studied, few studies have verified the production of CLA in vivo by CLA-producing bacteria. The variation in CLA production among strains of LAB depends on many factors, such as the intrinsic characteristics of each particular strain and the environmental conditions in which the strain grows.

In this context, the optimization and standardization of fermentation conditions are necessary to optimize CLA synthesis in functional foods. Many strains of *L. plantarum* have been proposed as human probiotics and are widely used as starter cultures to produce various fermented foods. Among the LAB, specific strains belonging to this species have been identified as the most efficient producers of CLA. Therefore, when fermenting foods, the use of appropriate strains of *L. plantarum* as a starter or additional culture should be considered as a critical step. Progress in research on the use of various strains of *L. plantarum* and the conditions for their maximum efficiency can serve as a key factor in the design of new CLA-enriched functional foods.

## Figures and Tables

**Table 1:** CLA production by *Lactiplantibacillus plantarum* (formerly *Lactobacillus plantarum*) in different culture media.

*L. plantarum* Strain	Culture Medium, Environmental Conditions	CLA Source	Total CLA	CLA Isomers (%)	Ref.
A6-1F	PBS, pH 7.0, 37 °C 15% (*w*/*v*) cell concentration	1.5 mg/mL LA	260.1–275.7 μg/mL	CLA1 mainly	[97]
AKU1009a	KPB, pH 6.5, 33% (wet *w*/*v*) washed cells, 108 h	0.12 mg/mL LA	40 mg/mL	38% CLA1 62% *t*9*t*11-CLA	[42]
ATCC8014	MRS, 40 °C, pH of 6.5, 48 h	0.1 mg/mL LA	37.5 μg/mL	expressed as CLA1	[98]
CRL1920 (CRL1935)	MRS, 37 °C, 48 h	0.5 mg/mL LA	17.3 μg/mL (17.5 μg/mL)	40% CLA1 30% CLA2 30% *t*9*t*11-CLA	[41]
DSM 20179	MRS (4.1% *v/v* inoculum, 4.0 g/L yeast extract), 37 °C, 24 h	3.0 mg/mL LA	240.7 μg/mL	90% CLA1 10% CLA2	[99]
HIF15	MRS + 0.05% L-cys-HCl, 37 °C, 48 h	0.5 mg/mL LA	46.2 μg/mL	75% CLA1 11% CLA2 13% *t*9*t*11-CLA	[95]
HIF15	SM, 10 mg/mL yeast extracts, 0.3% glucose	0.5 mg/mL LA	52.6 μg/mL	68% CLA1 9% CLA2 16% *t*9*t*11-CLA	[95]
JBCC105683	MRS, 30 °C 48 h	0.6 mg/mL LA	748.8 μg/mL	86% CLA1 14% CLA2	[75]
JBCC105675	MRS, 30 °C 48 h	0.2 mg/mL LA	427.2 μg/mL	85% CLA1 15% CLA2	[75]
JBNU105645	MRS, 30 °C 48 h	0.1 mg/mL LA	227.4 μg/mL	52% CLA1 48% CLA2	[75]
JCM 1551	0.1 M KPB, pH 6.5, 37 °C 12% (*w*/*v*) wet cell	3.4 mg/mL RA	2.4 mg/mL	21% CLA1 79% *t*9*t*11-CLA	[100]
L200	MRS, 30 °C, 48 h 2% inoculum	0.25 mg/mL LA + 0.1 mg/mL BSA	34.7 μg/mL	93% CLA1 2% CLA2 5% *t*9*t*11-CLA	[101]
L2C21E8 L3C1E8	MRS (or SM), 30 °C, 48 h	0.5 mg/mL LA	17.9 μg/mL 15.4 μg/mL	expressed as CLA1	[102]
lp15	MRS, 30 °C 48 h	0.1 mg/mL LA	26.1 μg/mL	76% CLA1 24% CLA2	[103]
*L. plantarum* from buffalo milk	MRS; 37 °C, pH of 5.5, 120 h	1.6 mg/mL LA with BSA (5 mg/mg LA)	272 μg/mL	51% CLA1 49% CLA2	[104]
NCUL005	MRS, 30 °C, 24 h	2.5 mL/L LA	623 μg/mL	32% CLA1 68% CLA2	[105]
WU-P19	MRS; 37 °C, pH of 6.0, 36 h (10% *v/v* inoculum) + chitosan	0.6 mg/mL LA	21 mg/g biomass	48% CLA1 52% CLA2	[106]
YW11	MRS, 24–36 h	10% (*v*/*v*) LA	n.d.	CLA1 (only with 10% LA) and other not conjugated LA metabolites	[107]
ZS2058	40 °C, pH of 6.5, 5 × 10^10^ CFU/mL	0.4 mg/mL LA	16.0 μg/(mL × h)	CLA1	[108]
ZS2058	MRS, 37 °C, 24 h	0.5 mg/mL LA	312.4 μg/mL	96.4% CLA1 3.6% CLA2	[109]
ZS2058	MRS, 37 °C, 48 h	0.55 mg/mL LA	0.313 mg/mL	66% CLA1 4.4% CLA2 29% *t*9*t*11-CLA	[110]

Abbreviations: BSA, bovine serum albumin; CLA1, *cis*-9, *trans*-11 CLA; CLA2, *trans-*10, *cis-*12 CLA; KPB, potassium phosphate buffer; LA, linoleic acid; MRS, de Man, Rogosa and Sharp medium; PBS, phosphate-buffered solution; RA, ricinoleic acid; SM, skim milk; n.d., not determined.

**Table 3:** CLA production by *Lactiplantibacillus plantarum* (formerly *Lactobacillus plantarum*) in different fermented foods.

*L. plantarum* Strain	Fermented Food, Environmental Conditions	CLA Source	Total CLA	CLA Isomers (%)	Ref.
AB20-961 (DSM2601)	sausage fermentation 79 h (73 h)	5% SAO	4.1 mg/g fat (7.5 mg/g fat) in sausage	n.d.	[129]
AB20-961 (DSM2601)	pH 7.9, 79 h (73 h) 8 log CFU/g fermented ground beef	5% FA source	7.9 mg/g fat (38.3 mg/g fat) in sausage	60% CLA1 40% CLA2	[96]
AB20-961	subculture in MRS, 37 °C, 24 h sucuk fermentation 12 h	2% HSO	6.1 mg/g fat in sucuk	69% CLA1 31% CLA2 in sucuk	[117]
HIF15	SM, 10 mg/mL yeast extracts, 0.3% glucose	0.5 mg/mL LA	52.6 μg/mL	68% CLA1 9% CLA2 16% *t*9*t*11-CLA	[95]
IMAU60042	soy milk, 42 °C, 48 h 2.0 × 10^7^ CFU/mL inoculum		122.4 μg/g	10% CLA1 90% CLA2	[131]
J25	SM, pH 6.4, 36 °C, 48 h 5% inoculum	2% LA	23.5 μg/mL	58% CLA1 42% CLA2	[156]
Lp in co-culture with *L. acidophilus*	SM, pH 6.4, 36 °C, 48 h 5% inoculum	5% SAO	316.5 μg/mL	n.d.	[132]
P1201	10% soy-powder milk, 37 °C, 48 h		198.7 μg/mL	90% CLA1	[157]
P1201	soy-powder hydrolyzed milk 35 °C, 48 h	1% SAO	1.3 mg/g	92% CLA1 8% CLA2	[134] [135]
S48	10% soy-powder milk, 37 °C, 48 h		183.6 μg/mL	90% CLA1	[157]
UALp-05 in co-culture with of *L. lactis* ssp. *lactis* and *L. lactis* ssp. *cremoris*	91% milk fat to produce cheddar cheese	9% SAO	75 μg/g in 90 days ripened cheese	25% CLA1 20% CLA2 20% *c*9*c*11-CLA 18% *t*9*c*11-CLA 16% *c*10*t*12-CLA	[43]

Abbreviations: CLA1, *cis*-9, *trans*-11 CLA; CLA2, *trans-*10, *cis-*12 CLA; FA, fatty acids; HSO, hydrolyzed sunflower oil; LA, linoleic acid; MRS, de Man, Rogosa and Sharp medium; SAO, safflower oil; SM, skim milk; n.d., not determined.

## Data Availability

The original contributions presented in the study are included in the article, further inquiries can be directed to the corresponding author.
